# Compositional alterations of gut microbiota in children with primary nephrotic syndrome after initial therapy

**DOI:** 10.1186/s12882-019-1615-4

**Published:** 2019-11-26

**Authors:** Yulin Kang, Dan Feng, Helen Ka-wai Law, Wei Qu, Ying Wu, Guang-hua Zhu, Wen-yan Huang

**Affiliations:** 1 Department of Nephrology and Rheumatology, Shanghai Children’s Hospital, Shanghai Jiao Tong University, Shanghai, 200062 China; 2 Department of Health Technology and Informatics, Faculty of Health and Social Science, Hong Kong Polytechnic University, Hunghom, Hong Kong, China

**Keywords:** Primary nephrotic syndrome, Gut microbiota, Glucocorticoids, Children

## Abstract

**Background:**

Primary nephrotic syndrome (PNS) is a common glomerular disease in children. T cell dysfunction plays a crucial role in the pathogenesis of PNS. Moreover, dysbiosis of gut microbiota contributes to immunological disorders. Whether the initial therapy of PNS affects gut microbiota remains an important question. Our study investigated compositional changes of gut microbiota after initial therapy.

**Methods:**

Fecal samples of 20 children with PNS were collected before and after 4-week initial therapy. Total bacteria DNA were extracted and the V3-V4 regions of bacteria 16S ribosomal RNA gene were sequenced. The composition of gut microbiota before and after initial therapy was analyzed by bioinformatics methods. The function of altered gut microbiota was predicted with PICRUSt method.

**Results:**

The richness and diversity of gut microbiota were similar before and after 4-week initial therapy. Gut microbiota at the phylum level was dominated by four phyla including *Firmicutes, Proteobacteria, Bacteroidetes* and *Actinobacteria*, but the increased relative abundance after initial therapy was found in *Deinococcus-Thermus* and *Acidobacteria*. At the genus level, the increased abundance of gut microbiota after initial therapy was observed in short chain fat acids (SCFA)-producing bacteria including *Romboutsia, Stomatobaculum* and *Cloacibacillus* (*p* < 0.05). Moreover, the predicted functional profile of gut microbiota showed that selenocompound metabolism, isoflavonoid biosynthesis and phosphatidylinositol signaling system weakened after initial therapy of PNS.

**Conclusions:**

Initial therapy of PNS increased SCFA-producing gut microbiota, but might diminish selenocompound metabolism, isoflavonoid biosynthesis and phosphatidylinositol signaling system in children.

## Background

Primary nephrotic syndrome (PNS) is a common glomerular disease in children, characterized by gross proteinuria, hypoalbuminenia, hyperlipidemia and edema [1]. T cell dysfunction plays a crucial role in PNS by producing cytokines that damage glomerular epithelial cells (podocytes) [2]. For instance, the imbalance of regulatory T cells (Treg cells) and T-helper17 cells (Th17 cells) is involving in the pathogenesis of minimal change nephrotic syndrome (MCNS) [3, 4]. These two subsets of lymphocytes play opposite roles, in which Treg cells have anti-inflammatory effects and maintain tolerance to self-antigen. In addition, Treg cells decrease in children with onset PNS, and they elevate with remission [5, 6]. However, the underlying reasons for these observations remain unclear. Recently, it has been known that dysbiosis of gut microbiota contributes to immunological disorders [7]. Therefore, analyzing gut microbiota may help to understand the pathophysiology of PNS in children.

Gut microbiota is a complex ecological community. Human gut harbors 100 trillion microbial cells, and the collection of microbial genome contains 100 times more genes than the human genome. Bacteroidetes, firmicutes and actinobacteria are predominant bacteria groups. Gut microbiota such as indigenous *clostridium* species induces the differentiation of Treg cells due to the microbe-derived butyrate which is one of short chain fat acids (SCFAs) [8]. In addition, the proportion of butyric-producing bacteria decreased significantly in children with relapsing PNS [9]. Taken together, it has been suggested that compositional alteration of gut microbiota regulates Treg cells and affects the outcome of PNS.

Aside from medications, the composition of gut microbiota can be influenced by age, gender, race, diet and host genetics [10–13]. The 2012 KDIGO Clinical Practice Guideline for Glomerulonephritis recommends that the initial therapy in children with PNS is oral prednisone for 4–6 weeks. Subsequently, patients receive alternate-day prednisone tapering in 2–5 months if initial therapy brings about remission [14]. 80–90% of children with PNS achieve complete remission with corticosteroid therapy, but 80–90% of them relapse [14, 15]. The long-term complications of steroid therapy include osteoporosis, infection and Cushing syndrome. Thus, calcium supplement is used to prevent glucocorticoids (GCs)-induced osteoporosis in children [16]. It is well known that patients with PNS achieve remission after GCs treatment from the anti-inflammatory and immunosuppressive effects. GCs induce genomic transcription of anti-inflammatory genes via cytosolic GC receptors, while large dosage of GCs activates non-genomic mechanisms [17]. Therefore, current research on the efficacy of GC is mainly focused on the glucocorticoid receptors. Nonetheless, it remains unknown whether gut microbiota changes after initial therapy in children with PNS. By investigating the compositional alteration of gut microbiota after initial therapy, we hope to shed new lights on developing new therapeutic approaches and preventing GC-associated side effects.

In our study, fecal samples were collected from children with PNS before and after 4-week initial therapy. Our results firstly showed that initial therapy of PNS in children altered the compositional of gut microbiota and might diminished the selenocompound metabolism, isoflavonoid biosynthesis and phosphatidylinositol signaling system.

## Methods

### Study cohort

The study was approved by the ethical committee of Shanghai Children's Hospital (#R037-F01). We recruited 20 children with PNS who were hospitalized between September 2016 and October 2017. Fecal samples were firstly collected from children at onset of PNS before treatment (Group A). Then, fecal samples were collected when these patients achieved complete remission after 4-week initial therapy (Group B). These patients have taken prednisone (2 mg/kg/day, maximum dosage was 60 mg/day) and compound of vitamin D3 and calcium carbonate (one tablet/day, containing 300 mg calcium and 100 Unit vitamin D3) orally for 4 weeks.

Inclusion criteria: Children were diagnosed with PNS and achieved complete remission after 4-week initial therapy. Complete remission was defined as urine protein: creatinine ratio (uPCR) < 200 mg/g for 3 consecutive days in accordance with 2012 KDIGO Clinical Practice Guideline for Glomerulonephritis [14].We excluded PNS patients who had concomitant diseases, estimated glomerular filtration rate (eGFR) < 90 ml/min/1.73m^2^, a history of gastrointestinal surgery, received antibiotic, probiotic and immunosuppressant treatment in the previous 2 months. Meanwhile, we collected clinical data including age, gender, delivery type, history of feeding types, eGFR, serum albumin, time to proteinuria resolution and the ratio of CD4+ to CD8 + T cells.

### Fecal samples collection and genomic DNA extraction

Fresh fecal samples were collected and stored in − 80 °C refrigerator until use. Total bacteria DNA were extracted by using QIAamp DNA stool Mini Kit (Qiagen, Hilden, Germany) as previously described [18]. The DNA concentration and purity were measured with a NanoDrop2000 spectrophotometer (Thermo Scientific, USA), and the integrity was evaluated by agarose gel electrophoresis.

### Preparation of 16S rRNA gene amplicon libraries and sequencing

The V3-V4 regions of bacteria 16S ribosomal RNA gene was amplified with the forward primer illumina adapter sequence1+ (5′-CCTACGGGNGGCWGCAG-3′) and reverse primer illumina adapter sequence2+ (5′-GACTACHVGGGTATCTAATCC-3′) as reported before [19]. PCR was performed in triplicate using a Gene Amp PCR-System 9700 (Applied Biosystems, Foster City, CA, USA) in a total volume of 25 μl, which contained 2.5 μl of 10× PCR buffer II, 0.5 unit of HerclueaseII DNA Polymerase High Fidelity (Agilent, USA), 0.4 μM of each primer, and 10 ng of template DNA. Thermal cycling conditions were as follows: an initial denaturation at 95 °C for 2 min, followed by 25 cycles at 95 °C for 20 s, 55 °C for 20 s, 72 °C for 45 s, and a final extension at 72 °C for 3 min. The quality of amplification products were checked using gel electrophoresis and were purified using the Agencourt AMPure XP Kit (Beckman Coulter, CA, USA). The sequencing of 16S rRNA gene amplicon was performed with the 2 × 250 bp paired-end method by using the Illumina MiSeq Bench top Sequencer [20]. The V3-V4 regions of 16S rRNA gene of fecal samples from 20 patients were sequenced. However, one sample from Group B was excluded as it failed to meet with the standard of sequencing experiment. A median depth of sequencing of 118828 reads per sample (80913–351500) was performed. All samples were sequenced by Genesky Biotechnologies Inc. (Shanghai, China).

### Bioinformatics analysis

Sample size was estimated by analyzing Species Accumulation Curve, and indicated that the 20 subjects met with the sample size calculation. Pairs of reads from the original DNA fragments were merged by using FLASH software (v1.2.11). The raw reads were checked with the QIIME quality filters under the default settings for Illumina processing. The qualified reads were chimera checked compared with the gold.fa database (http://drive5.com/uchime/gold.fa) and clustered into operational taxonomic units (OTUs) by UPARSE pipeline with 97% similarity cutoff value. The OTUs were classified based on Ribosomal Database Project (RDP) Release 9,201,203 and alpha diversity including Chao1, ACE, Shannon, Simpson, InvSimpson and coverage index were analyzed by using Mothur software. Principal Coordinate Analysis (PCoA) using Bray-Curtis distance, Jaccard, unweighted and weighted UniFrac metric was performed with R Project (Vegan package, V3.3.1). Compositional changes of gut microbiota at different taxonomic levels were analyzed by using Metastats method. Microbial metagenome functional information was inferred from 16S rRNA gene data by the PICRUSt (Phylogenetic Investigation of Communities by Reconstruction of Unobserved States) software using an extended ancestral-state reconstruction algorithm. The workflow of PICRUSt consists of gene content inference and metagenome inference. In brief, the OUTs were normalized upon 16 s rRNA gene copy number. Then, microbial community metagenomes were inferred and categorized into Kyoto Encyclopedia of Genes and Genomes (KEGG) pathways after the normalized OUT-table was input [21, 22]. The bioinformatics analysis was performed by Genesky Biotechnologies Inc.(Shanghai, China).

## Results

### Clinical and demographic characteristics of PNS patients

Twenty children (male: female = 15:5) with PNS were enrolled in this study. All of them were Han Chinese ethnicity. Age of onset was 3.5 ± 2.1 years old. Nine patients were born by vaginal delivery and 11 by caesarean section. As for the patterns of infant feeding, the number of patients receiving formula feeding, breast feeding, formula and breast feeding were 3,12 and 5 respectively. Serum albumin at onset was 16.2 ± 4.1 g/L and the ratio of CD4+/CD8+ T cells was 1.8 ± 0.6. Patients achieved remission in 13.6 ± 5.3 days after initial therapy. All had normal renal function (eGFR 207.0 ± 52.9 ml/1.73m^2^.min).

### The gut microbiota was altered in children with PNS

**Fig. 1:**
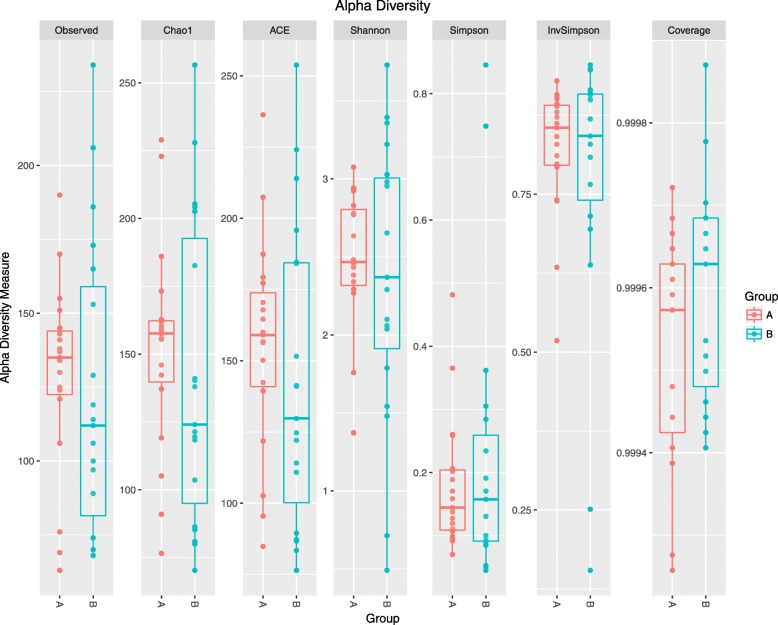
The richness and diversity of gut microbiota in children with primary nephrotic syndrome (PNS) before and after initial therapy**.** Alpha diversity of gut microbiota was reflected by the observed operational taxonomic units (OTUs), Chao1,ACE,Shannon,Simpson, InvSimpson and Coverage index. No significant differences were found in these indices after initial therapy (*p* > 0.05). Group A, B represented the samples from patients before and after initial therapy respectively

**Fig. 2:**
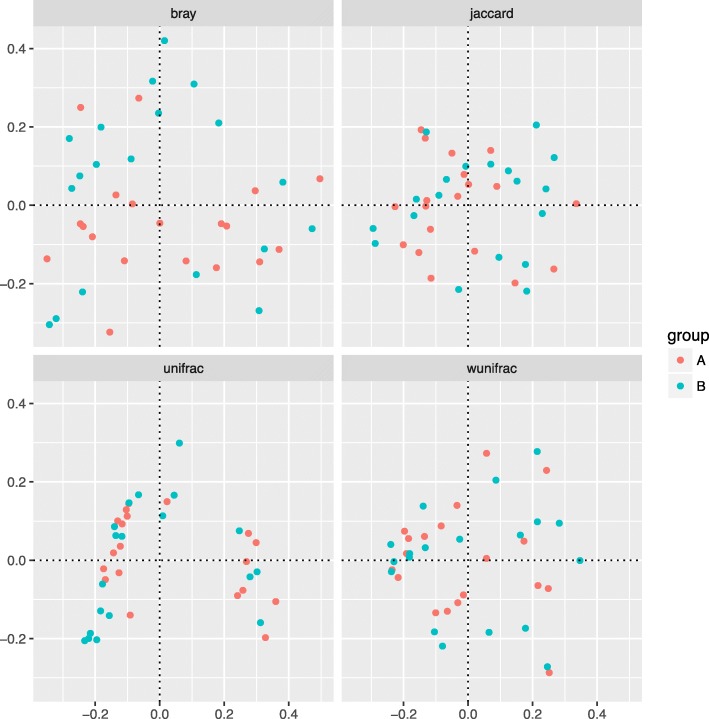
Principal coordinate analysis (PCoA) of gut microbiota based on OTUs**.** Phylogenetic tree-based distances between gut microbial communities of individuals were analyzed by using Bray-Curtis distance, Jaccard, unweighted and weighted UniFrac metric. No significant differences existed in the distances of fecal microbial community before and after initial therapy (*p* > 0.05). Each dot represents for one sample. Group A, B represented the samples from patients before and after initial therapy respectively. Abbreviations: bray, Bray-Curtis distance. Unifrac, unweighted UniFrac metric. Wunifrac, weighted UniFrac metric

A total of 577 distinct OTUs were observed. The observed OTUs, Chao1 and ACE indexes were used for evaluating microbial richness, while the Shannon, Simpson, InvSimpson and Coverage index were the indicators of microbial diversity. It showed that the richness and diversity of gut microbiota were similar between Group A and B (Fig. 1). Since feeding type may influence microbiota composition directly [23], we analyzed the differences in composition of gut microbiota in three different groups (formula feeding, breasting feeding, formula and breast feeding group) before initial therapy. As showed in Additional file 1, the richness and diversity of gut microbiota among these three groups before initial therapy were similar. The results of the beta diversity included Bray-Curtis, Jaccard, unweighted and weighted UniFrac distances. Principal Coordinate Analysis (PCoA) created a scatter plot to show the phylogenetic tree-based distances between gut microbiota of individuals. Unweighted UniFrac qualitatively measures the inter-individual differences with or without each taxon, while weighted UniFrac quantitatively analyses inter-individual differences in the relative abundance of each taxon. The data on Fig. 2 revealed that fecal microbial community did not differ significantly in patients with PNS before and after initial therapy.

**Fig. 3:**
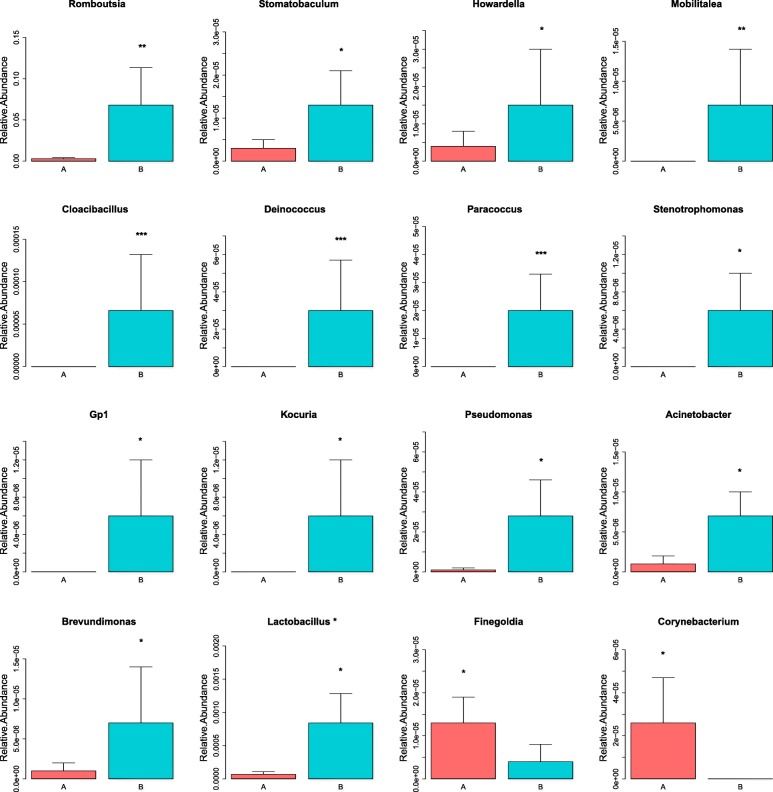
Compositional changes of gut microbiota at genus level**.** Sixteen significant differential genera were identified by using Metastats method. Relative abundance of the 16 genera was compared between Group A and B. *p < 0.05; **p < 0.01; ***p < 0.001. Group A, B represented the samples from patients before and after initial therapy respectively

To explore the fecal microbial changes after initial therapy, the composition of gut microbiota at taxonomic levels was analyzed. At the phylum level, *Firmicutes, Proteobacteria, Bacteroidetes* and *Actinobacteria* dominated in the gut microbiota, but the increased relative abundance after initial therapy was observed in *Deinococcus-Thermus* and *Acidobacteria* (Additional file 2 and Additional file 3). As showed in Fig. 3, increased relative abundance of microbe at the genus level were listed as follows: *Romboutsia, Stomatobaculum, Cloacibacillus,Howardella,Mobilitalea, Deinococcus, Paracoccus, Stenotrophomonas, Gp1, Kocuria, Pseudomonas, Acinetobacter, Brevundimonas* and *Lactobacillusbacteria*. However, lower relative abundance of *Finegoldia* and *Corynebacterium* was found after initial therapy. The raw data are available in the Additional file 4.

**Fig. 4:**
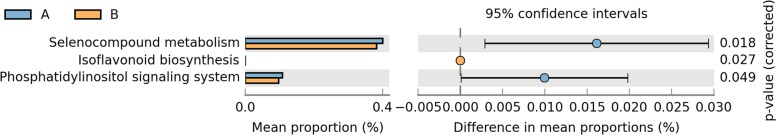
The predicted functional profile of gut microbiota before and after initial therapy. Microbial metagenome functional information was inferred from 16S rRNA gene data by the PICRUSt method. Three microbial metabolic pathways were weakened significantly after initial therapy (*p* < 0.05). Group A, B represented the samples from patients before and after initial therapy respectively

To explore the possible microbial metabolic pathways, the functional profile of gut microbiota was analyzed by the PICRUSt method. As showed in Fig. 4, microbial metabolic pathways including selenocompound metabolism, isoflavonoid biosynthesis and phosphatidylinositol signaling system significantly weakened after initial therapy.

## Discussion

Children with PNS have poor prognosis if remission is not achieved after initial therapy. Immune disorder takes part in pathogenesis of PNS [2]. Gut dysbiosis is capable of disturbing immunology systemically [24]. To the best of our knowledge, our study is the first to show that initial therapy altered the composition of gut microbiota in children with PNS. It might point the way in developing new therapeutic approaches by harnessing gut microbiota.

Our results showed that there were no changes in the richness and diversity of gut microbiota before and after initial therapy in children with PNS. Similar results were observed in dogs which received prednisolone for 14 days [25]. However, a reduction in the richness and diversity of microbiota has been reported in rats after dexamethasone (DEX) sodium phosphate treatment for 7 weeks [26]. The inconsistent results may be attributed to different types of GCs, study subjects and treatment time.

Although the sample size of this study is small, our data showed that gut microbiota was altered at different taxonomic levels after initial therapy. Our results showed that the phylum *Deinococcus-Thermus* and *Acidobacteria* increased after initial therapy, while no significant change was found in other commonly reported taxa such as *Firmicutes*, *Bacteroidetes* after GCs treatment. Diverse results were found in prednisolone- or DEX-treated animals. For instance, the prednisolone-treated mice showed a decreased relative abundance of *Bacteroidetes* and an increase in *Firmicutes* at the phylum level. Furthermore, the genus *Clostridium* sensu stricto decreased after 14 days of prednisolone treatment [27]. Additionally, after 7 weeks of DEX treatment, the relative abundances of *Firmicutes, Bacteroidetes, α-proteobacteria, γ- proteobacteria,* and *Actinobacteria* decreased in rat [26]. It has also been reported that crystallized corticosterone led to a reduction of potentially beneficial bacteria from the phylum *Firmicutes* in a wild bird (yellow-legged gull) [28]. Taken together, it suggested that GCs could disrupt gut microbiota. The fact that data were not consistent might be attributed to different kinds of GCs or different research models. In addition, the medications of initial therapy in PNS included the compound of vitamin D3 and calcium carbonate. *Lactococcus* significantly enriched in adults treated with vitamin D3 for 12 weeks, and calcium supplementation could also increase the numbers of intestinal microbiota such as *Ruminococcaceae,Akkermansia* and *Turicibacter* [29, 30]. Therefore, the combination of prednisone, compound of Vitamin D3 and calcium carbonate might have a synergistic effect on gut microbiota in patients with PNS.

Short-chain fatty acids (SCFA) are a group of fatty acids that are produced by the gut microbiota during the fermentation of partially and nondigestible polysaccharides. Our study showed that *Romboutsia, Stomatobaculum* and *Cloacibacillus* increased after initial therapy.These three genera were SCFA-producing bacteria [31–34]. The most well known SCFAs are acetate, propionate and butyrate. Butyrate and propionate induce the differentiation of colonic Treg cells which suppress effector T cells, resulting in tolerance to self-antigens. It needs to be verified whether increased SCFA-producing microbiota after initial therapy is associated with complete remission of PNS. Certainly, the function of other altered gut microbiota after initial therapy is worth of further investigation.

Three microbial metabolic pathways including selenocompound metabolism, isoflavonoid biosynthesis and phosphatidylinositol signaling system were significantly weakened after initial therapy. Many selenocompounds such as selenoproteins are key enzymes for maintaining the cellular redox homeostasis. Selenium and entailed selenoprotein deficiency lead to compromised immune responses [35]. Selenium-deficient diet also results in higher urinary protein in rats with puromycin aminonucleoside-induced nephrotic syndrome [36]. Selenocompound metabolism being weakened after initial therapy in children with PNS highlighted the possibility that less selenocompounds might be degraded. Additionally, we know that high-dose prednisone treatment increases serum selenium which improves antioxidant defense [37]. Thus, diminished selenocompound metabolism might help to keep an appropriate level of selenoproteins and contribute to remission of PNS after initial therapy.

Isoflavonoid is a group of water-soluble flavones that are antioxidants. Genistein (a major isoflavone of soybean) alleviates kidney injury in experimental nephrotic syndrome by improving renal antioxidant status [38]. Thus, the decreased isoflavonoid biosynthesis may be detrimental to kidney after initial therapy in PNS. It is also well known that phosphoinositides, the phosphorylated forms of phosphatidylinositol (PI), play important roles in cellular activities including lipid signaling, cell signaling and membrane trafficking. Hence, it is harmful to the recovery of nephrotic syndrome in the long term if phosphatidylinositol signaling system weakened in patients with PNS after initial therapy. Taken together, it is worthwhile to verify whether diminished selenocompound metabolism contributed to remission of PNS after initial therapy, while weakened isoflavonoid biosynthesis and phosphatidylinositol signaling are associated with the high rate of relapse occurs in children with PNS.

There are also some limitations in our study. Firstly, sample size was small even though it met with the sample size calculation. Multicenter investigations involving a large cohort of patients are needed. Secondly, the compositional alteration of gut microbiota was attributed to initial therapy which was a combined treatment. Thus, the changes of gut microbiota after single medication treatment such as prednisone or immunosuppressive agent would be the focus of future studies. Meanwhile, we are going to analyze metabolites of changed gut flora and verify their roles in remission of PNS. It would be meaningful in developing new therapeutic strategies for PNS if we are able to identify and culture specific microbiota species which could induce remission.

## Conclusions

Initial therapy of PNS increased SCFA-producing gut microbiota, but might diminish selenocompound metabolism, isoflavonoid biosynthesis and phosphatidylinositol signaling system in children. Our data were preliminary and the association between clinical outcome and altered gut microbiota needs to be confirmed in future. If altered gut microbiota affects the long-term outcome of PNS in children, a potentially useful and important avenue of treatment is just beginning.

## Supplementary information

**Additional file 1: **The microbial richness and diversity in patients with PNS before initial therapy. Microbial richness was reflected by the observed OTUs, Chao1 and ACE index, while microbial diversity was indicated by the Shannon, Simpson, InvSimpson and Coverage index. No significant differences were found in these indices among these three groups (formula feeding, breasting feeding, formula and breast feeding group) before initial therapy (*p* > 0.05).

**Additional file 2: **Compositional changes of gut microbiota at the phylum level. Metastats method was used to analyze compositional changes of gut microbiota at phylum level. Two significant differential phyla were identified. Relative abundance of the 2 phyla was compared between Group A and B. *p < 0.05; ***p < 0.001. Group A, B represented the groups of patients before and after initial therapy respectively.

**Additional file 3: **The profile of all identified phyla before and after initial therapy. Totally 13 phyla were identified by using Metastats method. Two phyla (*Deinococcus-Thermus* and *Acidobacteria*) increased significantly. “A_mean” and “B_mean” represented the relative abundance of gut microbiota in Group A and Group B respectively. *p* < 0.05 was considered statistically significant.

**Additional file 4: **The profile of all identified genera before and after initial therapy. Totally 150 genera were identified by using Metastats method. Fourteen genera (*Romboutsia, Stomatobaculum, Cloacibacillus,Howardella,Mobilitalea, Deinococcus, Paracoccus, Stenotrophomonas, Gp1, Kocuria, Pseudomonas, Acinetobacter, Brevundimonas*and *Lactobacillusbacteria*) increased significantly, while two genera (*Finegoldia* and *Corynebacterium*) decreased significantly. “A_mean” and “B_mean” represented the relative abundance of gut microbiota in Group A and Group B respectively. *p* < 0.05 was considered statistically significant.

## Data Availability

All data generated or analyzed during this study are included in this published article and its supplementary information files.

## Abbreviations

DEX: Dexamethasone
eGFR: Estimated glomerular filtration rate
GCs: Glucocorticoids
GR: Glucocorticoid receptor
KEGG: Kyoto Encyclopedia of Gene and Genomes
PCoA: Principal Coordinate Analysis
PI: Phosphatidylinositol
PNS: Primary nephrotic syndrome
SCFA: Short-chain fatty acid
SNP: single nucleotide polymorphism
Th17 cells: T-helper17 cells
Treg cells: Regulatory T cells
