# Supplementary figures and images for: Compositional alterations of gut microbiota in children with primary nephrotic syndrome after initial therapy
Source: BMC Nephrol. 2019 Nov 26;20:434. doi: 10.1186/s12882-019-1615-4 (PMC6878711; doi:10.1186/s12882-019-1615-4)

**Observed**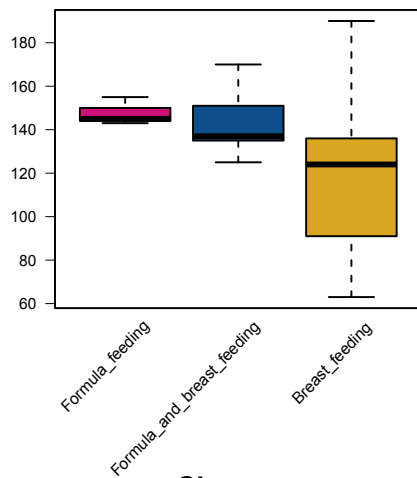**Chao1**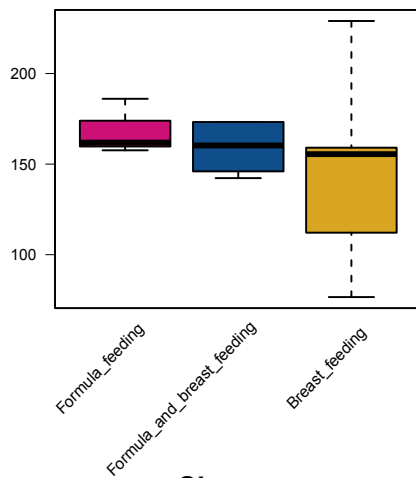**ACE**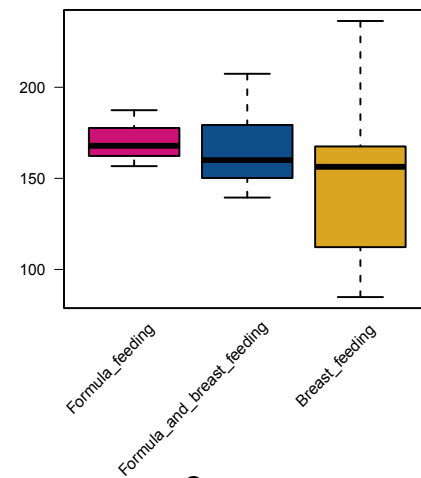**Shannon**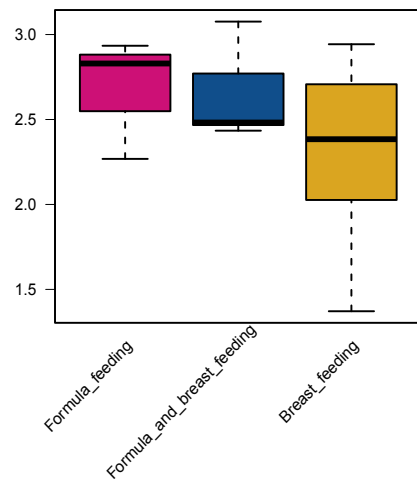**Simpson**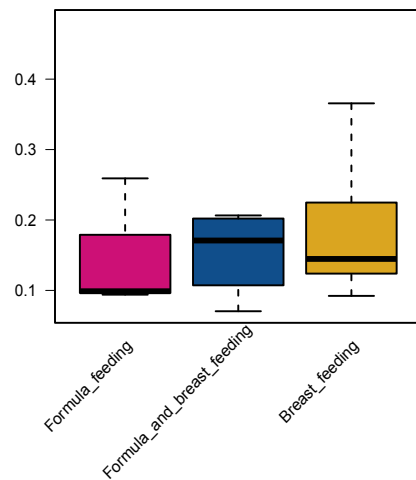**Coverage**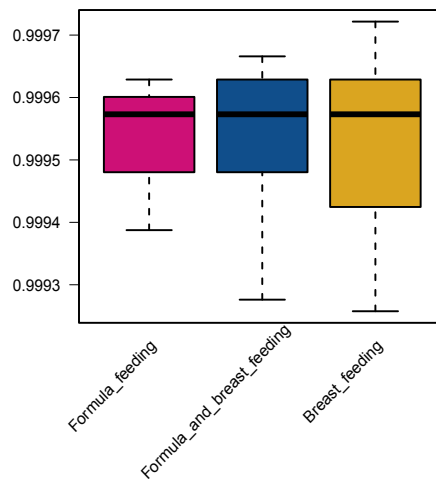

Supplement: Supplementary file 1 — Additional file 1: The microbial richness and diversity in patients with PNS before initial therapy. Microbial richness was reflected by the observed OTUs, Chao1 and ACE index, while microbial diversity was indicated by the Shannon, Simpson, InvSimpson and Coverage index. No significant differences were found in these indices among these three groups (formula feeding, breasting feeding, formula and breast feeding group) before initial therapy (p > 0.05). [file 12882_2019_1615_MOESM1_ESM.pdf]

### Deinococcus-Thermus

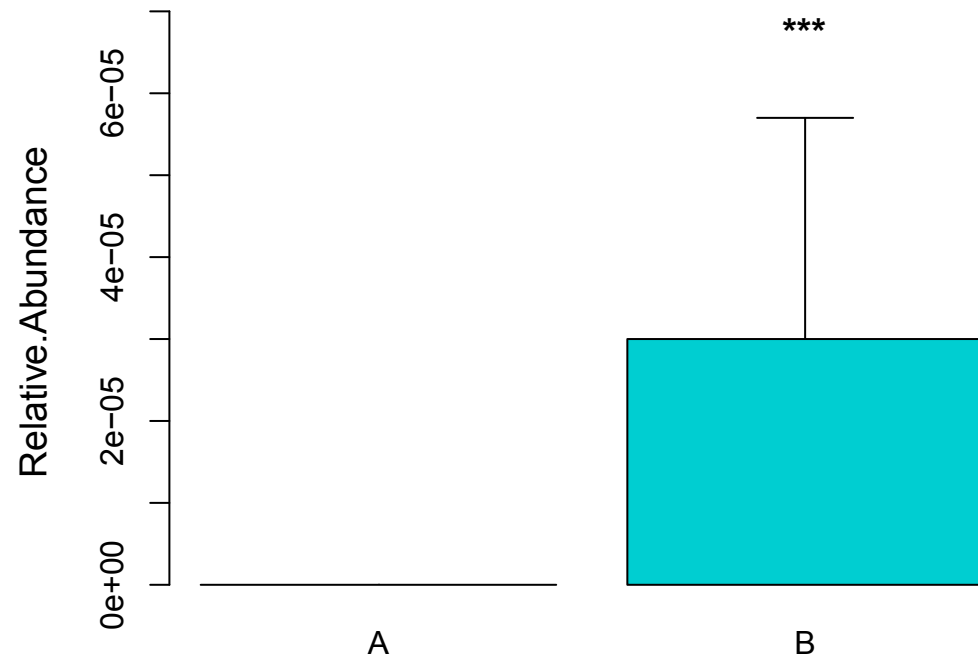

### Acidobacteria

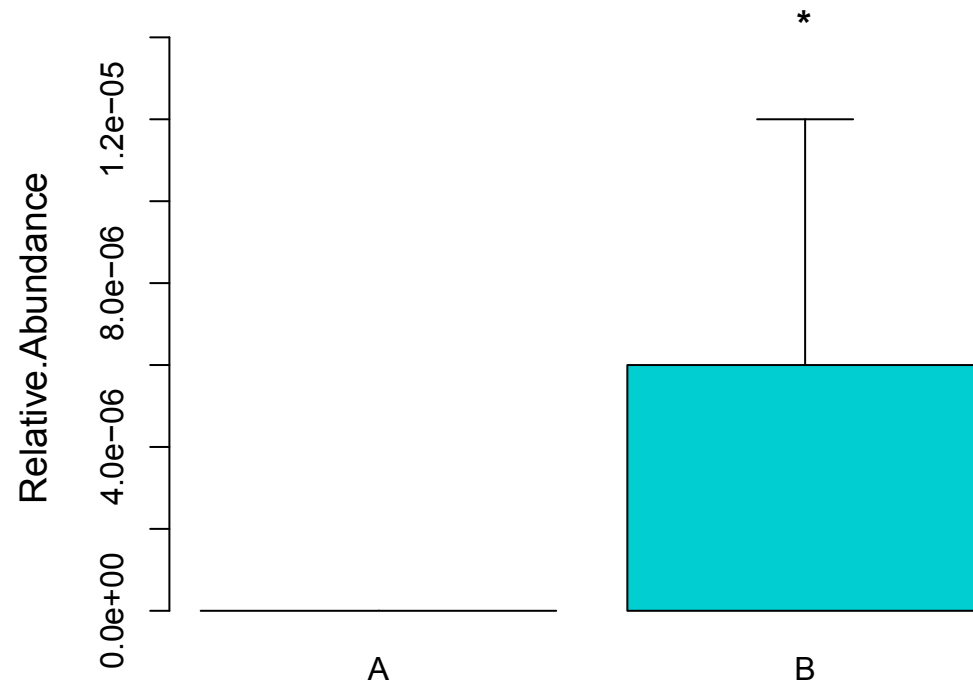

Supplement: Supplementary file 2 — Additional file 2: Compositional changes of gut microbiota at the phylum level. Metastats method was used to analyze compositional changes of gut microbiota at phylum level. Two significant differential phyla were identified. Relative abundance of the 2 phyla was compared between Group A and B. *p < 0.05; ***p < 0.001. Group A, B represented the groups of patients before and after initial therapy respectively. [file 12882_2019_1615_MOESM2_ESM.pdf]
